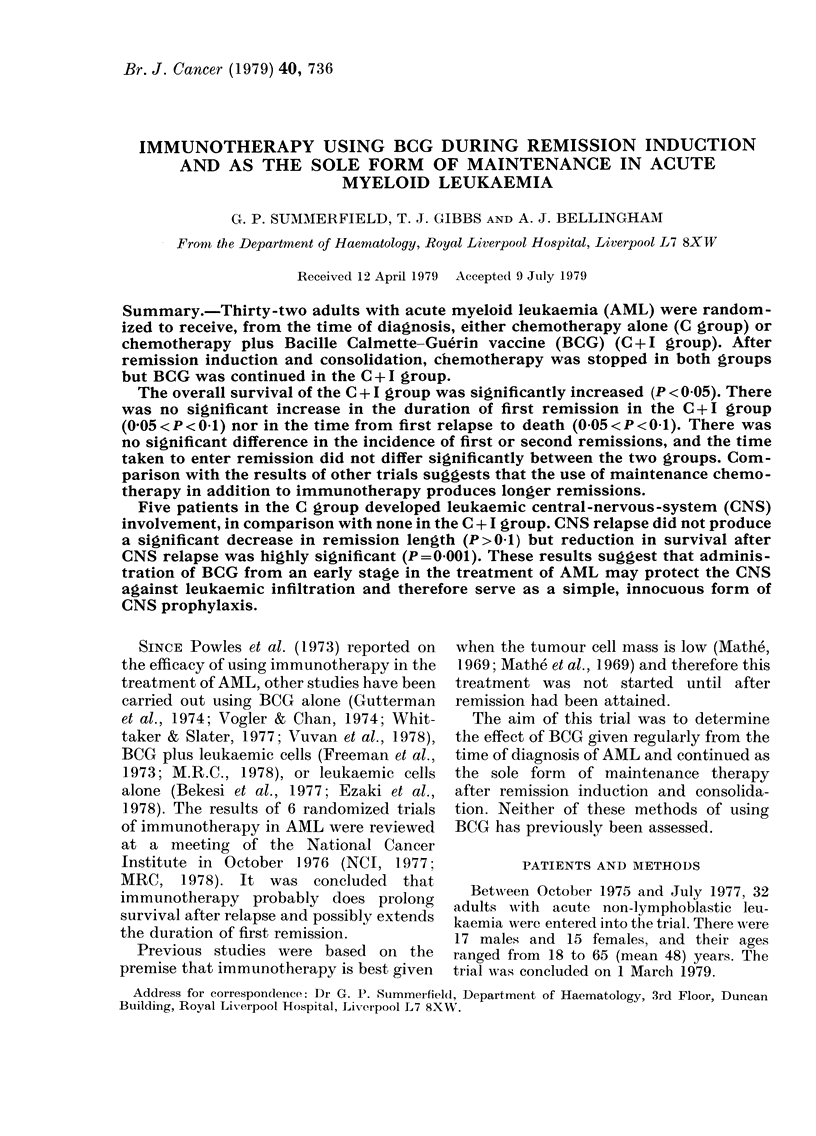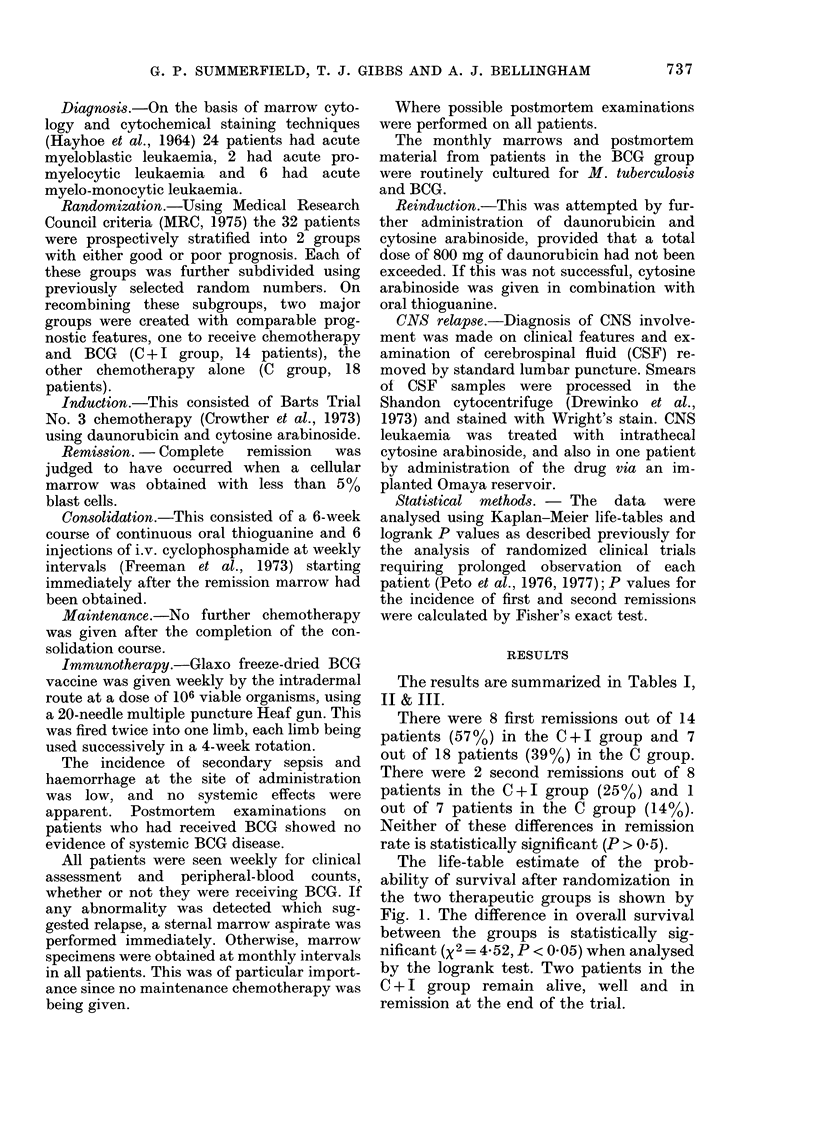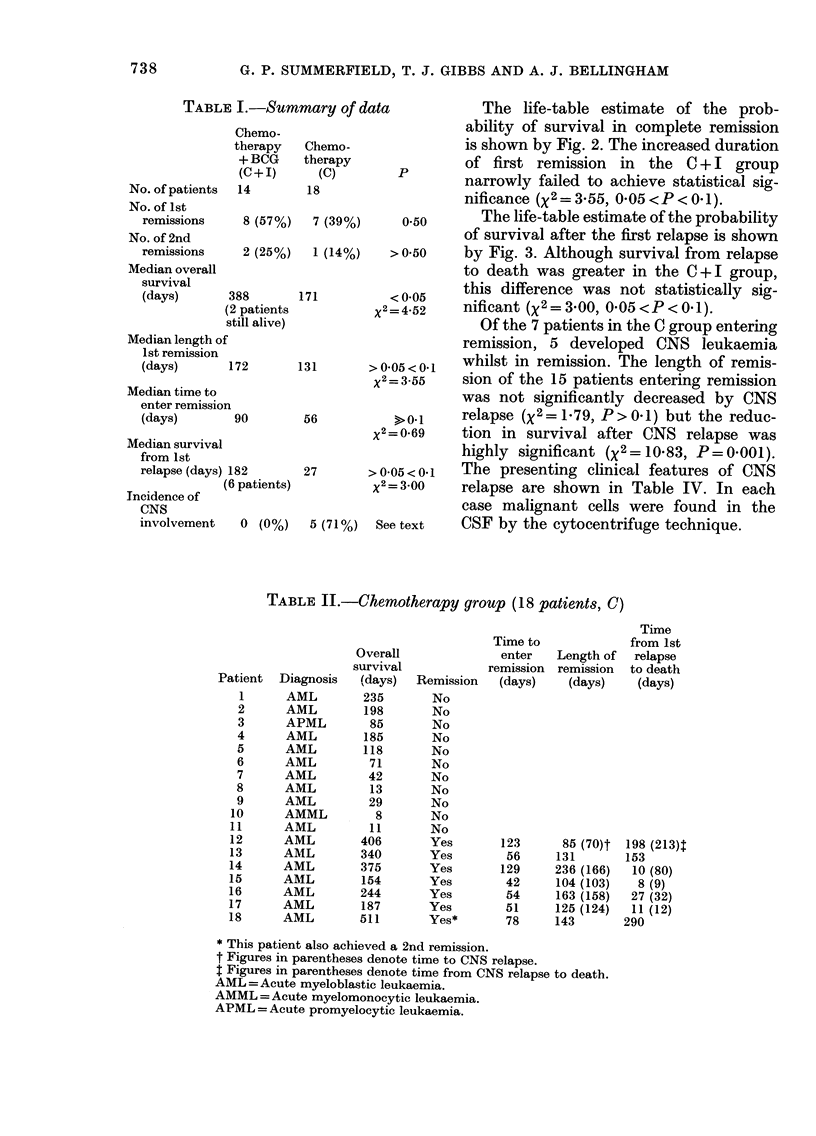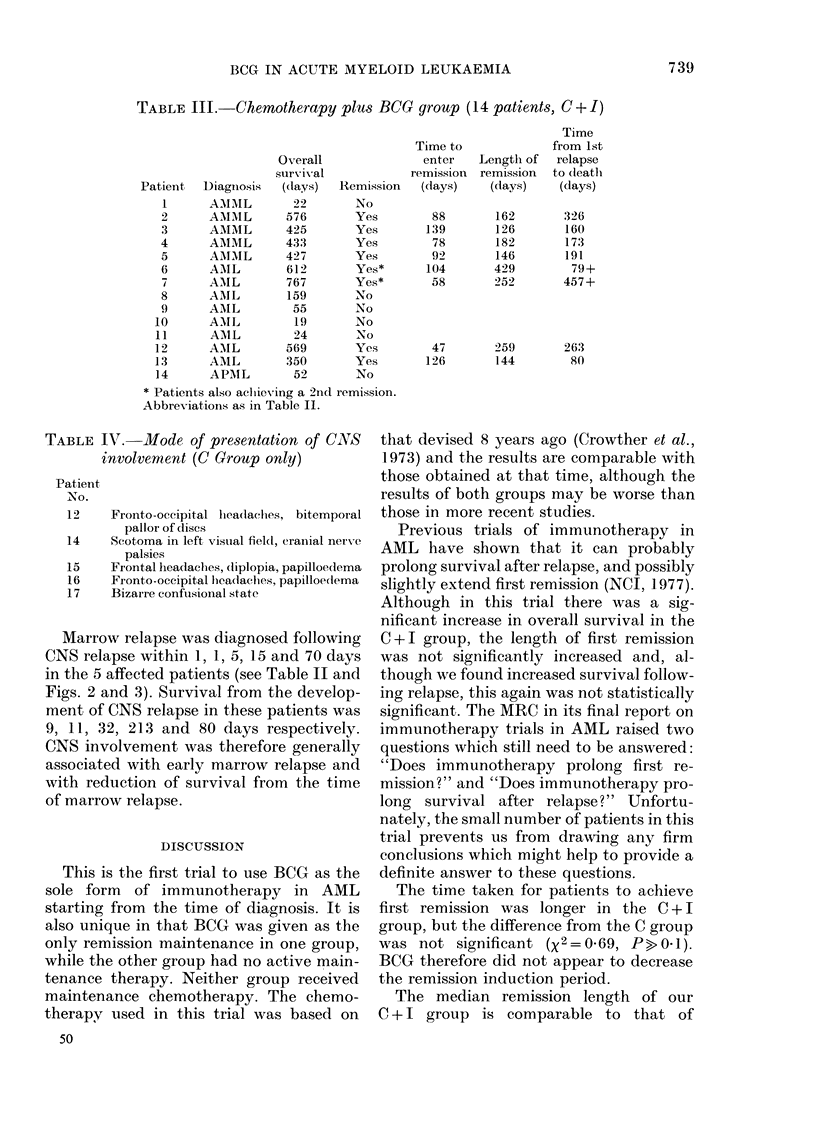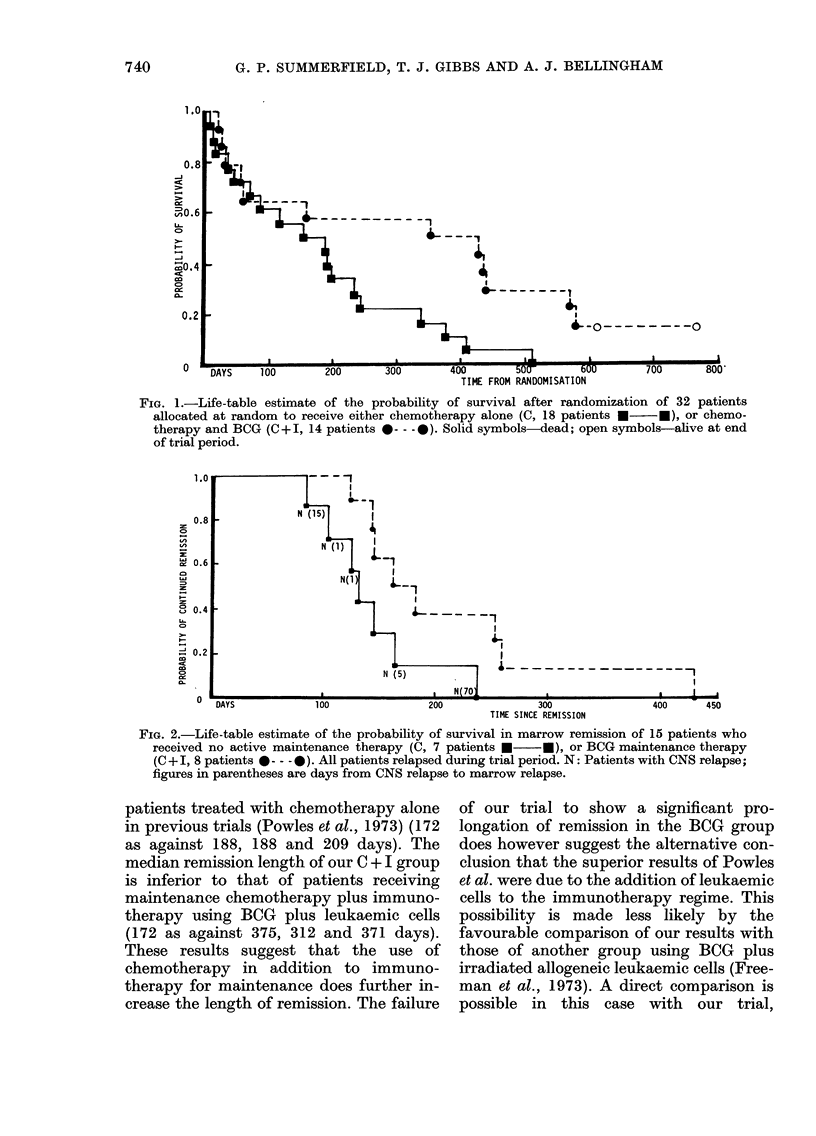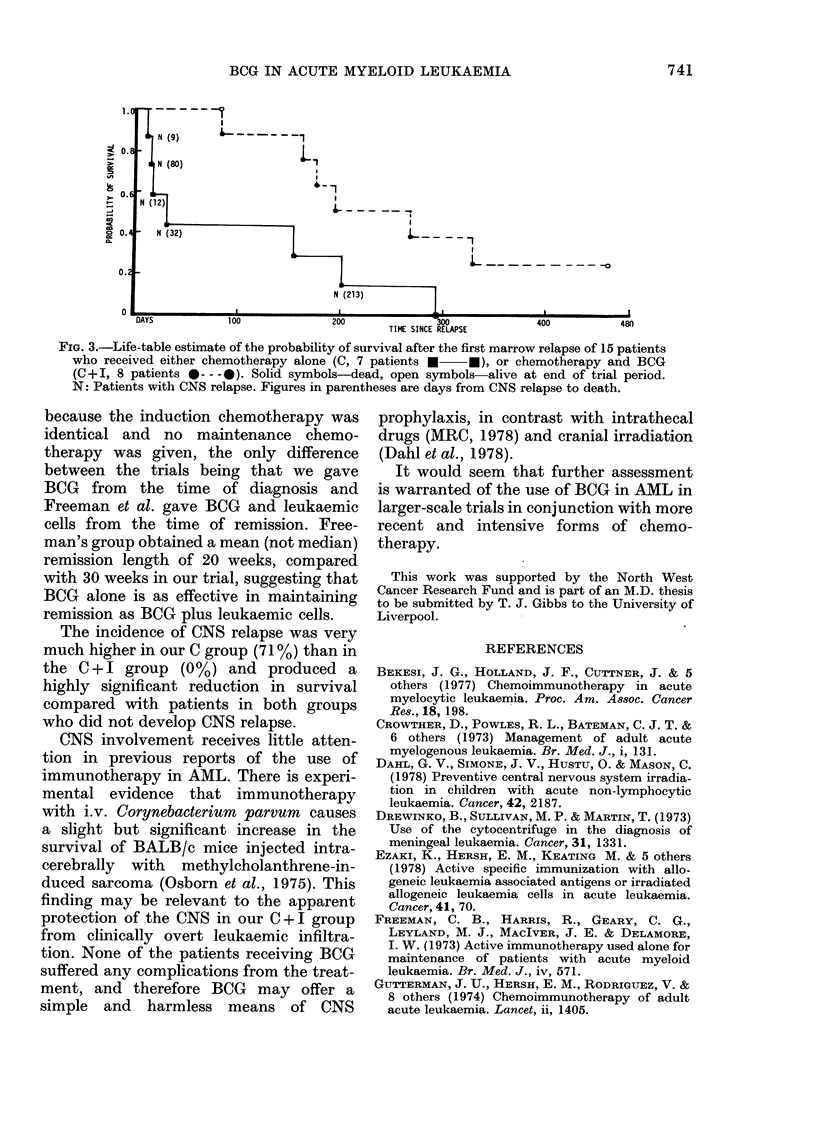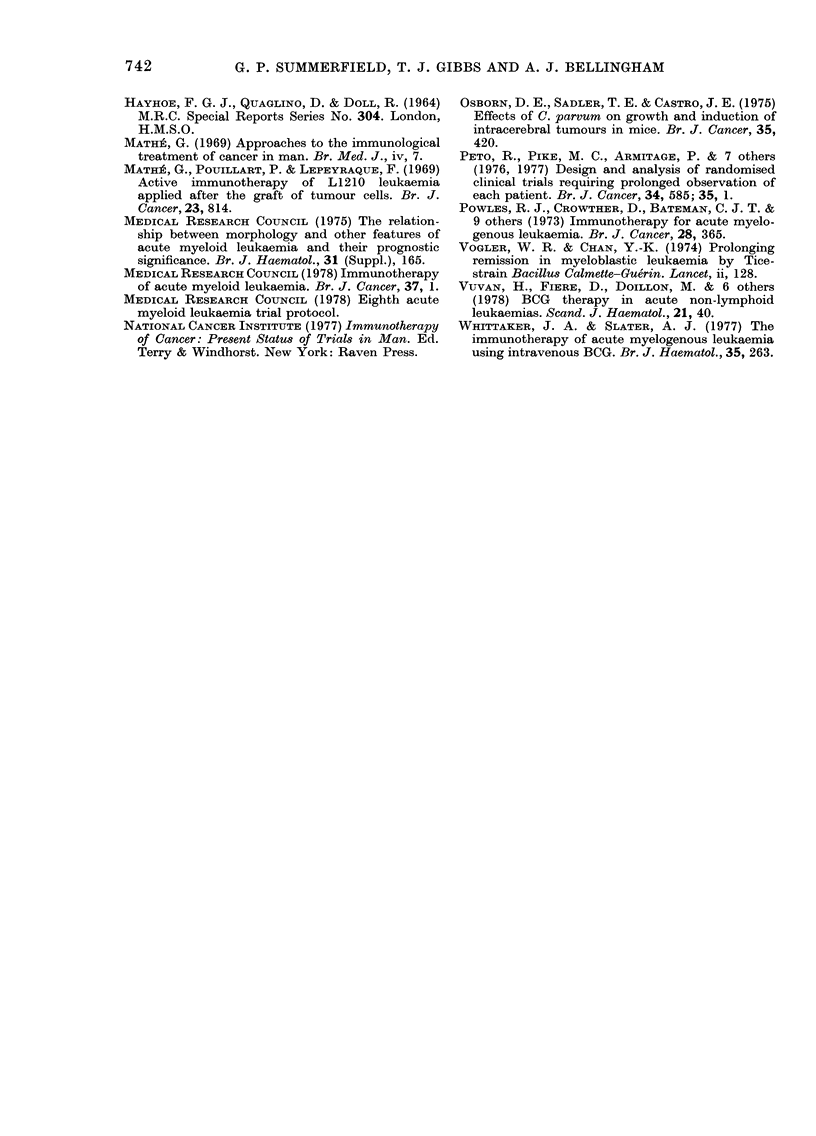# Immunotherapy using BCG during remission induction and as the sole form of maintenance in acute myeloid leukaemia.

**DOI:** 10.1038/bjc.1979.254

**Published:** 1979-11

**Authors:** G. P. Summerfield, T. J. Gibbs, A. J. Bellingham

## Abstract

Thirty-two adults with acute myeloid leukaemia (AML) were randomized to receive, from the time of diagnosis, either chemotherapy alone (C group) or chemotherapy plus Bacille Calmette-Guérin vaccine (BCG) (C+I group). After remission induction and consolidation, chemotherapy was stopped in both groups but BCG was continued in the C+I group. The overall survival of the C+I group was significantly increased (P less than 0.05). There was no significant increase in the duration of first remission in the C+I group (0.05 less than P less than 0.1) nor in the time from first relapse to death (0.05 less than P less than 0.1). There was no significant difference in the incidence of first or second remissions, and the time taken to enter remission did not differ significantly between the two groups. Comparison with the results of other trials suggests that the use of maintenance chemotherapy in addition to immunotherapy produces longer remissions. Five patients in the C group developed leukaemic central-nervous-system (CSN) involvement, in comparison with none in the C+I group. CNS relapse did not produce a significant decrease in remission length (P greater than 0.1) but reduction in survival after CNS relapse was highly significant (P = 0.001). These results suggest that administration of BCG from an early stage in the treatment of AML may protect the CNS against leukaemic infiltration and therefore serve as a simple, innocuous form of CNS prophylaxis.


*Br. J. Cancer* (1979) **40**, 736

### IMMUNOTHERAPY USING BCG DURING REMISSION INDUCTION AND AS THE SOLE FORM OF MAINTENANCE IN ACUTE MYELOID LEUKAEMIA

G. P. SUMMERFIELD, T. J. GIBBS AND A. J. BELLINGHAM

*From the Department of Haematology, Royal Liverpool Hospital, Liverpool L7 8XW*

Received 12 April 1979 Accepted 9 July 1979

**Summary.—Thirty-two adults with acute myeloid leukaemia (AML) were randomized to receive, from the time of diagnosis, either chemotherapy alone (C group) or chemotherapy plus Bacille Calmette–Guérin vaccine (BCG) (C+I group). After remission induction and consolidation, chemotherapy was stopped in both groups but BCG was continued in the C+I group.**

**The overall survival of the C+I group was significantly increased ($P<0{\cdot}05$). There was no significant increase in the duration of first remission in the C+I group ($0{\cdot}05<P<0{\cdot}1$) nor in the time from first relapse to death ($0{\cdot}05<P<0{\cdot}1$). There was no significant difference in the incidence of first or second remissions, and the time taken to enter remission did not differ significantly between the two groups. Comparison with the results of other trials suggests that the use of maintenance chemotherapy in addition to immunotherapy produces longer remissions.**

**Five patients in the C group developed leukaemic central-nervous-system (CNS) involvement, in comparison with none in the C+I group. CNS relapse did not produce a significant decrease in remission length ($P>0{\cdot}1$) but reduction in survival after CNS relapse was highly significant ($P=0{\cdot}001$). These results suggest that administration of BCG from an early stage in the treatment of AML may protect the CNS against leukaemic infiltration and therefore serve as a simple, innocuous form of CNS prophylaxis.**

SINCE Powles *et al.* (1973) reported on the efficacy of using immunotherapy in the treatment of AML, other studies have been carried out using BCG alone (Gutterman *et al.*, 1974; Vogler & Chan, 1974; Whittaker & Slater, 1977; Vuvan *et al.*, 1978), BCG plus leukaemic cells (Freeman *et al.*, 1973; M.R.C., 1978), or leukaemic cells alone (Bekesi *et al.*, 1977; Ezaki *et al.*, 1978). The results of 6 randomized trials of immunotherapy in AML were reviewed at a meeting of the National Cancer Institute in October 1976 (NCI, 1977; MRC, 1978). It was concluded that immunotherapy probably does prolong survival after relapse and possibly extends the duration of first remission.

Previous studies were based on the premise that immunotherapy is best given when the tumour cell mass is low (Mathé, 1969; Mathé *et al.*, 1969) and therefore this treatment was not started until after remission had been attained.

The aim of this trial was to determine the effect of BCG given regularly from the time of diagnosis of AML and continued as the sole form of maintenance therapy after remission induction and consolidation. Neither of these methods of using BCG has previously been assessed.

### PATIENTS AND METHODS

Between October 1975 and July 1977, 32 adults with acute non-lymphoblastic leukaemia were entered into the trial. There were 17 males and 15 females, and their ages ranged from 18 to 65 (mean 48) years. The trial was concluded on 1 March 1979.

Address for correspondence: Dr G. P. Summerfield, Department of Haematology, 3rd Floor, Duncan Building, Royal Liverpool Hospital, Liverpool L7 8XW.

*Diagnosis.*—On the basis of marrow cytology and cytochemical staining techniques (Hayhoe *et al.*, 1964) 24 patients had acute myeloblastic leukaemia, 2 had acute promyelocytic leukaemia and 6 had acute myelo-monocytic leukaemia.

*Randomization.*—Using Medical Research Council criteria (MRC, 1975) the 32 patients were prospectively stratified into 2 groups with either good or poor prognosis. Each of these groups was further subdivided using previously selected random numbers. On recombining these subgroups, two major groups were created with comparable prognostic features, one to receive chemotherapy and BCG (C+I group, 14 patients), the other chemotherapy alone (C group, 18 patients).

*Induction.*—This consisted of Barts Trial No. 3 chemotherapy (Crowther *et al.*, 1973) using daunorubicin and cytosine arabinoside.

*Remission.* — Complete remission was judged to have occurred when a cellular marrow was obtained with less than 5% blast cells.

*Consolidation.*—This consisted of a 6-week course of continuous oral thioguanine and 6 injections of i.v. cyclophosphamide at weekly intervals (Freeman *et al.*, 1973) starting immediately after the remission marrow had been obtained.

*Maintenance.*—No further chemotherapy was given after the completion of the consolidation course.

*Immunotherapy.*—Glaxo freeze-dried BCG vaccine was given weekly by the intradermal route at a dose of $10^6$ viable organisms, using a 20-needle multiple puncture Heaf gun. This was fired twice into one limb, each limb being used successively in a 4-week rotation.

The incidence of secondary sepsis and haemorrhage at the site of administration was low, and no systemic effects were apparent. Postmortem examinations on patients who had received BCG showed no evidence of systemic BCG disease.

All patients were seen weekly for clinical assessment and peripheral-blood counts, whether or not they were receiving BCG. If any abnormality was detected which suggested relapse, a sternal marrow aspirate was performed immediately. Otherwise, marrow specimens were obtained at monthly intervals in all patients. This was of particular importance since no maintenance chemotherapy was being given.

Where possible postmortem examinations were performed on all patients.

The monthly marrows and postmortem material from patients in the BCG group were routinely cultured for *M. tuberculosis* and BCG.

*Reinduction.*—This was attempted by further administration of daunorubicin and cytosine arabinoside, provided that a total dose of 800 mg of daunorubicin had not been exceeded. If this was not successful, cytosine arabinoside was given in combination with oral thioguanine.

*CNS relapse.*—Diagnosis of CNS involvement was made on clinical features and examination of cerebrospinal fluid (CSF) removed by standard lumbar puncture. Smears of CSF samples were processed in the Shandon cytocentrifuge (Drewinko *et al.*, 1973) and stained with Wright's stain. CNS leukaemia was treated with intrathecal cytosine arabinoside, and also in one patient by administration of the drug *via* an implanted Omaya reservoir.

*Statistical methods.* — The data were analysed using Kaplan–Meier life-tables and logrank $P$ values as described previously for the analysis of randomized clinical trials requiring prolonged observation of each patient (Peto *et al.*, 1976, 1977); $P$ values for the incidence of first and second remissions were calculated by Fisher's exact test.

### RESULTS

The results are summarized in Tables I, II & III.

There were 8 first remissions out of 14 patients (57%) in the C+I group and 7 out of 18 patients (39%) in the C group. There were 2 second remissions out of 8 patients in the C+I group (25%) and 1 out of 7 patients in the C group (14%). Neither of these differences in remission rate is statistically significant ($P > 0{\cdot}5$).

The life-table estimate of the probability of survival after randomization in the two therapeutic groups is shown by Fig. 1. The difference in overall survival between the groups is statistically significant ($\chi^2 = 4{\cdot}52$, $P < 0{\cdot}05$) when analysed by the logrank test. Two patients in the C+I group remain alive, well and in remission at the end of the trial.

TABLE I.—*Summary of data*

| | Chemotherapy +BCG (C+I) | Chemotherapy (C) | P |
|---|---|---|---|
| No. of patients | 14 | 18 | |
| No. of 1st remissions | 8 (57%) | 7 (39%) | 0·50 |
| No. of 2nd remissions | 2 (25%) | 1 (14%) | >0·50 |
| Median overall survival (days) | 388 (2 patients still alive) | 171 | <0·05 $\chi^2=4{\cdot}52$ |
| Median length of 1st remission (days) | 172 | 131 | >0·05<0·1 $\chi^2=3{\cdot}55$ |
| Median time to enter remission (days) | 90 | 56 | ⩾0·1 $\chi^2=0{\cdot}69$ |
| Median survival from 1st relapse (days) | 182 (6 patients) | 27 | >0·05<0·1 $\chi^2=3{\cdot}00$ |
| Incidence of CNS involvement | 0 (0%) | 5 (71%) | See text |

The life-table estimate of the probability of survival in complete remission is shown by Fig. 2. The increased duration of first remission in the C+I group narrowly failed to achieve statistical significance ($\chi^2=3{\cdot}55$, $0{\cdot}05<P<0{\cdot}1$).

The life-table estimate of the probability of survival after the first relapse is shown by Fig. 3. Although survival from relapse to death was greater in the C+I group, this difference was not statistically significant ($\chi^2=3{\cdot}00$, $0{\cdot}05<P<0{\cdot}1$).

Of the 7 patients in the C group entering remission, 5 developed CNS leukaemia whilst in remission. The length of remission of the 15 patients entering remission was not significantly decreased by CNS relapse ($\chi^2=1{\cdot}79$, $P>0{\cdot}1$) but the reduction in survival after CNS relapse was highly significant ($\chi^2=10{\cdot}83$, $P=0{\cdot}001$). The presenting clinical features of CNS relapse are shown in Table IV. In each case malignant cells were found in the CSF by the cytocentrifuge technique.

TABLE II.—*Chemotherapy group* (18 *patients*, *C*)

| Patient | Diagnosis | Overall survival (days) | Remission | Time to enter remission (days) | Length of remission (days) | Time from 1st relapse to death (days) |
|---|---|---|---|---|---|---|
| 1 | AML | 235 | No | | | |
| 2 | AML | 198 | No | | | |
| 3 | APML | 85 | No | | | |
| 4 | AML | 185 | No | | | |
| 5 | AML | 118 | No | | | |
| 6 | AML | 71 | No | | | |
| 7 | AML | 42 | No | | | |
| 8 | AML | 13 | No | | | |
| 9 | AML | 29 | No | | | |
| 10 | AMML | 8 | No | | | |
| 11 | AML | 11 | No | | | |
| 12 | AML | 406 | Yes | 123 | 85 (70)† | 198 (213)‡ |
| 13 | AML | 340 | Yes | 56 | 131 | 153 |
| 14 | AML | 375 | Yes | 129 | 236 (166) | 10 (80) |
| 15 | AML | 154 | Yes | 42 | 104 (103) | 8 (9) |
| 16 | AML | 244 | Yes | 54 | 163 (158) | 27 (32) |
| 17 | AML | 187 | Yes | 51 | 125 (124) | 11 (12) |
| 18 | AML | 511 | Yes* | 78 | 143 | 290 |

* This patient also achieved a 2nd remission.
† Figures in parentheses denote time to CNS relapse.
‡ Figures in parentheses denote time from CNS relapse to death.
AML = Acute myeloblastic leukaemia.
AMML = Acute myelomonocytic leukaemia.
APML = Acute promyelocytic leukaemia.

TABLE III.—*Chemotherapy plus BCG group* (14 *patients*, $C+I$)

| Patient | Diagnosis | Overall survival (days) | Remission | Time to enter remission (days) | Length of remission (days) | Time from 1st relapse to death (days) |
|---|---|---|---|---|---|---|
| 1 | AMML | 22 | No | | | |
| 2 | AMML | 576 | Yes | 88 | 162 | 326 |
| 3 | AMML | 425 | Yes | 139 | 126 | 160 |
| 4 | AMML | 433 | Yes | 78 | 182 | 173 |
| 5 | AMML | 427 | Yes | 92 | 146 | 191 |
| 6 | AML | 612 | Yes* | 104 | 429 | 79+ |
| 7 | AML | 767 | Yes* | 58 | 252 | 457+ |
| 8 | AML | 159 | No | | | |
| 9 | AML | 55 | No | | | |
| 10 | AML | 19 | No | | | |
| 11 | AML | 24 | No | | | |
| 12 | AML | 569 | Yes | 47 | 259 | 263 |
| 13 | AML | 350 | Yes | 126 | 144 | 80 |
| 14 | APML | 52 | No | | | |

* Patients also achieving a 2nd remission.
Abbreviations as in Table II.

TABLE IV.—*Mode of presentation of CNS involvement* (*C Group only*)

| Patient No. | |
|---|---|
| 12 | Fronto-occipital headaches, bitemporal pallor of discs |
| 14 | Scotoma in left visual field, cranial nerve palsies |
| 15 | Frontal headaches, diplopia, papilloedema |
| 16 | Fronto-occipital headaches, papilloedema |
| 17 | Bizarre confusional state |

Marrow relapse was diagnosed following CNS relapse within 1, 1, 5, 15 and 70 days in the 5 affected patients (see Table II and Figs. 2 and 3). Survival from the development of CNS relapse in these patients was 9, 11, 32, 213 and 80 days respectively. CNS involvement was therefore generally associated with early marrow relapse and with reduction of survival from the time of marrow relapse.

### DISCUSSION

This is the first trial to use BCG as the sole form of immunotherapy in AML starting from the time of diagnosis. It is also unique in that BCG was given as the only remission maintenance in one group, while the other group had no active maintenance therapy. Neither group received maintenance chemotherapy. The chemotherapy used in this trial was based on that devised 8 years ago (Crowther *et al.*, 1973) and the results are comparable with those obtained at that time, although the results of both groups may be worse than those in more recent studies.

Previous trials of immunotherapy in AML have shown that it can probably prolong survival after relapse, and possibly slightly extend first remission (NCI, 1977). Although in this trial there was a significant increase in overall survival in the C+I group, the length of first remission was not significantly increased and, although we found increased survival following relapse, this again was not statistically significant. The MRC in its final report on immunotherapy trials in AML raised two questions which still need to be answered: "Does immunotherapy prolong first remission?" and "Does immunotherapy prolong survival after relapse?" Unfortunately, the small number of patients in this trial prevents us from drawing any firm conclusions which might help to provide a definite answer to these questions.

The time taken for patients to achieve first remission was longer in the C+I group, but the difference from the C group was not significant ($\chi^2 = 0{\cdot}69$, $P \gg 0{\cdot}1$). BCG therefore did not appear to decrease the remission induction period.

The median remission length of our C+I group is comparable to that of

FIG. 1.—Life-table estimate of the probability of survival after randomization of 32 patients allocated at random to receive either chemotherapy alone (C, 18 patients ■——■), or chemotherapy and BCG (C+I, 14 patients ●- - -●). Solid symbols—dead; open symbols—alive at end of trial period.

FIG. 2.—Life-table estimate of the probability of survival in marrow remission of 15 patients who received no active maintenance therapy (C, 7 patients ■——■), or BCG maintenance therapy (C+I, 8 patients ●- - -●). All patients relapsed during trial period. N: Patients with CNS relapse; figures in parentheses are days from CNS relapse to marrow relapse.

patients treated with chemotherapy alone in previous trials (Powles *et al.*, 1973) (172 as against 188, 188 and 209 days). The median remission length of our C + I group is inferior to that of patients receiving maintenance chemotherapy plus immunotherapy using BCG plus leukaemic cells (172 as against 375, 312 and 371 days). These results suggest that the use of chemotherapy in addition to immunotherapy for maintenance does further increase the length of remission. The failure of our trial to show a significant prolongation of remission in the BCG group does however suggest the alternative conclusion that the superior results of Powles *et al.* were due to the addition of leukaemic cells to the immunotherapy regime. This possibility is made less likely by the favourable comparison of our results with those of another group using BCG plus irradiated allogeneic leukaemic cells (Freeman *et al.*, 1973). A direct comparison is possible in this case with our trial,

FIG. 3.—Life-table estimate of the probability of survival after the first marrow relapse of 15 patients who received either chemotherapy alone (C, 7 patients ■——■), or chemotherapy and BCG (C+I, 8 patients ●- - -●). Solid symbols—dead, open symbols—alive at end of trial period. N: Patients with CNS relapse. Figures in parentheses are days from CNS relapse to death.

because the induction chemotherapy was identical and no maintenance chemotherapy was given, the only difference between the trials being that we gave BCG from the time of diagnosis and Freeman *et al.* gave BCG and leukaemic cells from the time of remission. Freeman's group obtained a mean (not median) remission length of 20 weeks, compared with 30 weeks in our trial, suggesting that BCG alone is as effective in maintaining remission as BCG plus leukaemic cells.

The incidence of CNS relapse was very much higher in our C group (71%) than in the C+I group (0%) and produced a highly significant reduction in survival compared with patients in both groups who did not develop CNS relapse.

CNS involvement receives little attention in previous reports of the use of immunotherapy in AML. There is experimental evidence that immunotherapy with i.v. *Corynebacterium parvum* causes a slight but significant increase in the survival of BALB/c mice injected intracerebrally with methylcholanthrene-induced sarcoma (Osborn *et al.*, 1975). This finding may be relevant to the apparent protection of the CNS in our C+I group from clinically overt leukaemic infiltration. None of the patients receiving BCG suffered any complications from the treatment, and therefore BCG may offer a simple and harmless means of CNS prophylaxis, in contrast with intrathecal drugs (MRC, 1978) and cranial irradiation (Dahl *et al.*, 1978).

It would seem that further assessment is warranted of the use of BCG in AML in larger-scale trials in conjunction with more recent and intensive forms of chemotherapy.

This work was supported by the North West Cancer Research Fund and is part of an M.D. thesis to be submitted by T. J. Gibbs to the University of Liverpool.

REFERENCES

BEKESI, J. G., HOLLAND, J. F., CUTTNER, J. & 5 others (1977) Chemoimmunotherapy in acute myelocytic leukaemia. *Proc. Am. Assoc. Cancer Res.*, **18**, 198.

CROWTHER, D., POWLES, R. L., BATEMAN, C. J. T. & 6 others (1973) Management of adult acute myelogenous leukaemia. *Br. Med. J.*, i, 131.

DAHL, G. V., SIMONE, J. V., HUSTU, O. & MASON, C. (1978) Preventive central nervous system irradiation in children with acute non-lymphocytic leukaemia. *Cancer*, **42**, 2187.

DREWINKO, B., SULLIVAN, M. P. & MARTIN, T. (1973) Use of the cytocentrifuge in the diagnosis of meningeal leukaemia. *Cancer*, **31**, 1331.

EZAKI, K., HERSH, E. M., KEATING M. & 5 others (1978) Active specific immunization with allogeneic leukaemia associated antigens or irradiated allogeneic leukaemia cells in acute leukaemia. *Cancer*, **41**, 70.

FREEMAN, C. B., HARRIS, R., GEARY, C. G., LEYLAND, M. J., MACIVER, J. E. & DELAMORE, I. W. (1973) Active immunotherapy used alone for maintenance of patients with acute myeloid leukaemia. *Br. Med. J.*, iv, 571.

GUTTERMAN, J. U., HERSH, E. M., RODRIGUEZ, V. & 8 others (1974) Chemoimmunotherapy of adult acute leukaemia. *Lancet*, ii, 1405.

HAYHOE, F. G. J., QUAGLINO, D. & DOLL, R. (1964) M.R.C. Special Reports Series No. **304**. London, H.M.S.O.

MATHÉ, G. (1969) Approaches to the immunological treatment of cancer in man. *Br. Med. J.*, iv, 7.

MATHÉ, G., POUILLART, P. & LEPEYRAQUE, F. (1969) Active immunotherapy of L1210 leukaemia applied after the graft of tumour cells. *Br. J. Cancer*, **23**, 814.

MEDICAL RESEARCH COUNCIL (1975) The relationship between morphology and other features of acute myeloid leukaemia and their prognostic significance. *Br. J. Haematol.*, **31** (Suppl.), 165.

MEDICAL RESEARCH COUNCIL (1978) Immunotherapy of acute myeloid leukaemia. *Br. J. Cancer*, **37**, 1.

MEDICAL RESEARCH COUNCIL (1978) Eighth acute myeloid leukaemia trial protocol.

NATIONAL CANCER INSTITUTE (1977) *Immunotherapy of Cancer: Present Status of Trials in Man.* Ed. Terry & Windhorst. New York: Raven Press.

OSBORN, D. E., SADLER, T. E. & CASTRO, J. E. (1975) Effects of *C. parvum* on growth and induction of intracerebral tumours in mice. *Br. J. Cancer*, **35**, 420.

PETO, R., PIKE, M. C., ARMITAGE, P. & 7 others (1976, 1977) Design and analysis of randomised clinical trials requiring prolonged observation of each patient. *Br. J. Cancer*, **34**, 585; **35**, 1.

POWLES, R. J., CROWTHER, D., BATEMAN, C. J. T. & 9 others (1973) Immunotherapy for acute myelogenous leukaemia. *Br. J. Cancer*, **28**, 365.

VOGLER, W. R. & CHAN, Y.-K. (1974) Prolonging remission in myeloblastic leukaemia by Tice-strain *Bacillus Calmette–Guérin*. *Lancet*, ii, 128.

VUVAN, H., FIERE, D., DOILLON, M. & 6 others (1978) BCG therapy in acute non-lymphoid leukaemias. *Scand. J. Haematol.*, **21**, 40.

WHITTAKER, J. A. & SLATER, A. J. (1977) The immunotherapy of acute myelogenous leukaemia using intravenous BCG. *Br. J. Haematol.*, **35**, 263.